# A Rare *KIF1A* Missense Mutation Enhances Synaptic Function and Increases Seizure Activity

**DOI:** 10.3389/fgene.2020.00061

**Published:** 2020-02-27

**Authors:** Yi Guo, Yuanyuan Chen, Min Yang, Xin Xu, Zijun Lin, Junhong Ma, Hongnian Chen, Yida Hu, Yuanlin Ma, Xuefeng Wang, Xin Tian

**Affiliations:** ^1^ Department of Neurology, The First Affiliated Hospital of Chongqing Medical University, Chongqing Key Laboratory of Neurology, Chongqing, China; ^2^ Center of Epilepsy, Beijing Institute for Brain Disorders, Beijing, China

**Keywords:** *KIF1A*, epilepsy, synaptic plasticity, dendritic spine, epileptogenesis

## Abstract

Although genetic factors are considered a main etiology of epilepsy, the causes of genetic epilepsy in the majority of epilepsy patients remain unknown. Kinesin family member 1A (KIF1A), a neuron-specific motor protein that moves along with microtubules, is responsible for the transport of membranous organelles and synaptic vesicles. Variants of *KIF1A* have recently been associated with hereditary spastic paraplegia (HSP), hereditary sensory and autonomic neuropathy type 2 (HSANII), and intellectual disability. However, mutations in *KIF1A* have not been detected in patients with epilepsy. In our study, we conducted customized sequencing of epilepsy-related genes of a family with six patients with generalized epilepsy over three generations and identified a rare heterozygous mutation (c.1190C > A, p. Ala397Asp) in *KIF1A*. Whole-cell recordings from primary cultured neurons revealed that the mutant *KIF1A* increases the excitatory synaptic transmission but not the intrinsic excitability of neurons, and phenotype testing in zebrafish showed that this rare mutation results in epileptic seizure-like activity. These results provide new evidence demonstrating that *KIF1A* dysfunction is involved in epileptogenesis.

## Introduction

Genetic factors are one of the main causes of epilepsy that have been confirmed by the International League Against Epilepsy (ILAE) (Berg et al., 2010; Scheffer et al., 2016). In 1995, Steinlein et al. demonstrated that missense mutations in *CHRNA4* can cause autosomal dominant nocturnal frontal lobe epilepsy (Steinlein et al., 1995). A survey of 1,258 patients with epilepsy (of which 958 had focal epilepsy) showed that common gene variants collectively explain at least 26% of the phenotypic variation among patients with all forms of epilepsy and 27% of that among patients with focal epilepsy (Speed et al., 2014). Miller et al. analyzed possible factors affecting epilepsy in twins and found that genetic factors play a significant role in various types of seizures (Miller et al., 1998). Genetic epilepsy is defined as a direct result of a known or presumed genetic defect, and recurrent seizures are the core symptom of these disorders (Scheffer et al., 2016). With the development of next-generation sequencing techniques, significant progress toward understanding the genetic mechanisms underlying the disease has recently been achieved. However, the genetic etiology of epilepsy in most patients remains unknown.

KIF1A is responsible for the transport of membranous organelles and synaptic vesicles in neurons, and mutations in *KIF1A* have been associated with hereditary spastic paraplegia (HSP), hereditary sensory and autonomic neuropathy type 2 (HSANII), and intellectual disability (Riviere et al., 2011; Citterio et al., 2015). Only a handful of previous reports have related *KIF1A* gene mutations to epileptic seizure, and thus far, the role of *KIF1A* in the origin of epilepsy has received limited attention.

In our study, we identified a rare mutation of *KIF1A* in a family with six patients over three generations who presented with generalized epilepsy. First, we found that the mutant KIF1A increased the excitatory synaptic density, and a functional test of zebrafish transfected with wild-type (WT) and mutant *kif1aa* using the tol2 vector showed that the mutation caused epileptic seizure-like behavior and epileptic electrophysiological activity.

## Materials and Methods

### Genetic Screening of Epilepsy Patients

DNA was extracted from peripheral blood lymphocytes collected from all individuals belonging to the family of interest. We performed customized sequencing of epilepsy-related genes (**Supplementary Table 1**) using NextSeq500 (Illumina, San Diego, CA, USA) to identify a possible causative gene mutation. Single nucleotide variations, insertions and deletions were then examined using GATK (https://software.broadinstitute.org/gatk/). Variants annotated in ANNOVAR (http://annovar.openbioinformatics.org/en/latest/) and variants with a minor allele frequency of at least 0.05 were filtered out (the candidate normal population database included 1000 Genomes, Exome Variant Server and EXAC). PolyPhen-2, SIFT and MutationTaster and GERP++ were used to predict the damage caused by all candidate variants, and the candidate causative variants obtained from the sequencing analysis were also confirmed by Sanger sequencing.

### Construction of Plasmids

Flag-tagged human WT *KIF1A* was obtained by subcloning the complementary DNA (cDNA) into the pcDNA3.1-3xFlag-T2A-EGFP plasmid using primers:

F: CTTGGTACCGAGCTCGGATCCGCCACCATGGCCGGGGCTTCGGTGAA
R: GAAGGGCCCTCTAGACTCGAGGACCCGCATCTGGGCAGACC

The missense mutation 1190C > A (p. Ala397Asp) was constructed by overlap PCR mutagenesis using the following primers:

KIF1A A397D-1F, CTTGGTACCGAGCTCGGATCCGCCACCATGGCCGGGGCTTCGGTGAA;
KIF1A A397D-1R, CCCACCAGGtCATTGGTCATGTCAGTGATGTCGC;
KIF1A A397D-2F, ATGACCAATGaCCTGGTGGGTATGAGCCCCTCAT; and
KIF1A A397D-2R, GAAGGGCCCTCTAGACTCGAGGACCCGCATCTGGGCAGACC.

### Culture and Transfection of Primary Hippocampal Neurons

The procedure used for culturing primary hippocampal neurons was previously described (Yang et al., 2019). Briefly, hippocampal neurons were prepared from C57BL/6 mouse embryos at gestational day 18. The brains were removed, and the hippocampi were dissected from the brains. The obtained tissue was digested using 3 ml of trypsin solution for 15 min. The obtained neurons were cultured in neurobasal medium supplemented with B27, L-glutamine, penicillin, and streptomycin. The neurons were plated onto glass coverslips coated with poly-D-lysine in a 37°C incubator with 5% CO_2_. The neurons were transfected with KIF1A-WT or KIF1A-A397D plasmids using the calcium phosphate transfection method at 7 days *in vitro* (DIV7).

### Whole-Cell Recordings

Whole-cell recordings were obtained from green fluorescent protein (GFP)-positive neurons transfected with KIF1A-WT or KIF1A-A397D plasmids. To explore the intrinsic excitability of the neurons, we applied a depolarizing current of 500 ms in the current clamp mode starting from −30 pA and at increments of 10 pA to induce action potentials. The rheobase was defined as the first current step that was able to induce action potential firing in a neuron. For the evaluation of synaptic transmission, we maintained the neurons at the potential of −70 mV in artificial cerebrospinal fluid (ACSF) containing 140 mM NaCl, 5 mM KCl, 1.8 mM CaCl_2_, 1.2 mM MgCl_2_, and 10 mM D-glucose. The miniature excitatory post-synaptic currents (mEPSCs) were recorded in the presence of 1 μM tetrodotoxin (TTX) and 0.1 mM picrotoxin (PTX), and the miniature inhibitory post-synaptic currents (mIPSCs) were recorded in the presence of 1 μM TTX, 10 μM 6-cyano-7-nitroquinoxaline-2,3-dione (CNQX), and 100 μM (2R)-amino-5-phosphonovaleric acid (APV). Electrophysiological data were acquired using a Multiclamp 700B amplifier and Digidata 1440A, and the data were analyzed using Mini Analysis (Synaptosoft, Leonia, NJ, USA) and Clampfit 10.3.

### Western Blot and Immunofluorescence Staining

For western blotting, total protein was obtained from primary cultured hippocampal neurons 3 days after transfection with the KIF1A-WT or KIF1A-A397D plasmid using the calcium phosphate transfection method at DIV7. The western blot analysis was performed as described previously (Zhang et al., 2019), antibodies using for western blot including: mouse anti-Flag (Sigma), Goat anti-mouse IgG (Proteintech), rabbit anti-GAPDH (Proteintech), Goat anti-rabbit IgG (Proteintech).

For immunofluorescence staining, the cultured neurons were fixed with 4% paraformaldehyde/4% sucrose in phosphate-buffered saline (PBS) for 40 min and permeabilized with 0.3% Triton X-100 in PBS for 15 min. Primary antibodies were diluted and added to the coverslip, and incubated overnight in a humidified chamber at 4°C, then coverslips were washed three times with PBS and incubated with the secondary fluorescent antibodies at room temperature for 1 h. Images were captured using a confocal laser scanning microscope (Leica, Wetzlar, Germany). The following primary antibodies were used: rabbit anti-GFP (Invitrogen), Guinea pig anti-vGLUT (synaptic systems), mouse anti-PSD-95 (Cell Signaling Technology), mouse anti-Flag (Sigma). The following secondary antibodies were used: Alexa Fluor 488-conjugated goat anti rabbit IgG (Invitrogen), Alexa Fluor 405-conjugated goat anti Guinea pig IgG (Jackson ImmunoResearch), Alexa Fluor 594-conjugated goat anti mouse IgG (Invitrogen).

For the assessment of neuronal morphology, the neurons were transfected with the KIF1A-WT or KIF1A-A397D plasmid at DIV7 and fixed at DIV10. In Sholl analysis, concentric circles were drawn around the soma every 10 μm, and the intersections with neurite branches were counted, then primary and secondary neurites in the same neurons were then counted.

For the analysis of spine density, the neurons were transfected with the KIF1A-WT or KIF1A-A397D plasmid at DIV7 and fixed at DIV16. The spine density was then quantified from two randomly selected secondary or tertiary dendrites per neuron. All these experiments were performed in a blinded manner by two observers.

### Zebrafish Maintenance and Breeding

Adult male and female zebrafish (*Danio rerio*) of the AB strain were obtained from the China Zebrafish Resource Center (http://www.zfish.cn/) and maintained at 28.5°C under a 14-h light/10-h dark cycle using standard procedures. The fertilized eggs were collected *via* natural spawning. The fertilized embryos were maintained in medium containing 1.5 mM 4-(2-hydroxyethyl)-1-piperazineethanesulfonic acid (HEPES), pH 7.6, 17.4 mM NaCl, 0.21 mM KCl, 0.12 mM MgSO_4_, and 0.18 mM Ca(NO3)_2_ at 28.5°C. All the experiments with zebrafish were approved by the Ethics Committee of Chongqing Medical University.

### Overexpression Experiments

WT zebrafish *kif1aa* cDNA was cloned into the Tol2 expression vector (a gift from Koichi Kawakami, National Institute of Genetics) using following primers:

F: AAAGAATTCCTCGACGGATCCGCCACCatggcaggggcctcggtg;
R: TCACCATGGTGGCGACCGGTCCAGAGCCTCCACCCCCaaacctcatctgcccagc;

The mutation encoding A433D in zebrafish was cloned into the *kif1aa* sequence *via* PCR site-directed mutation using primers: F: gacaaaagcccttcctactacact; R: tagtaggaagggcttttgtctatttgagtaatctgctgtatcctg. Embryos at the one-cell stage were transfected with the WT *kif1aa*-Tol2 or A433D *kif1aa*-Tol2 plasmid with transposase *via* cytoplasmic microinjection.

### Behavior Monitoring and Local Field Potential Recording

For behavior monitoring, zebrafish larvae at 5 days post-fertilization (d.p.f.) were placed in 24-well Falcon culture dishes that contained embryo medium. A charge-coupled device (CCD) camera and EthoVision 3.1 locomotion tracking software were used to monitor the swim activity of the mutant (A433D *kif1aa*-Tol2) and WT (WT *kif1aa*-Tol2) larvae, and each larva was monitored for 15 min. The swim activity was categorized by three observers who were blind to the larva phenotype as stage 0 (baseline activity), stage 1 (small increase in swim activity), and stage 2 (large increase in movement) (Hortopan et al., 2010).

For local field potential recording, zebrafish larvae at 5 d.p.f. were immobilized in low-melting temperature agarose. A glass filled with 2 M NaCl was placed in the optic tecum of zebrafish larvae, and each recording was performed for 15 min in the current clamp mode with high-pass filtering above 0.1 Hz and low-pass filtering below 1 kHz. The digital gain was 10 [MultiClamp 700B Amplifier (Axon, Sunnyvale, CA, USA) and Digidata1440A (Axon, Sunnyvale, CA, USA)]. Spontaneous events were defined as instances in which the amplitude exceeded three times the background noise (Schubert et al., 2014). Clampfit 10.3 (Molecular Devices, Sunnyvale, CA, USA) software was used for the data analyses.

### Statistical Analysis

The measurement data (means ± SDs) from two groups were compared using Student's *t* test, and the data from more than two parameters were analyzed by two-way ANOVA. Chi-square tests were used to compare the differences in the behavior and local field potential (LFP) of larvae. *P* < 0.05 was considered statistically significant, and the statistical analyses were performed using GraphPad Prism version 7.0 (GraphPad Software).

## Results

### Clinical Characteristics of Patients in the Family

**Figure 1:**
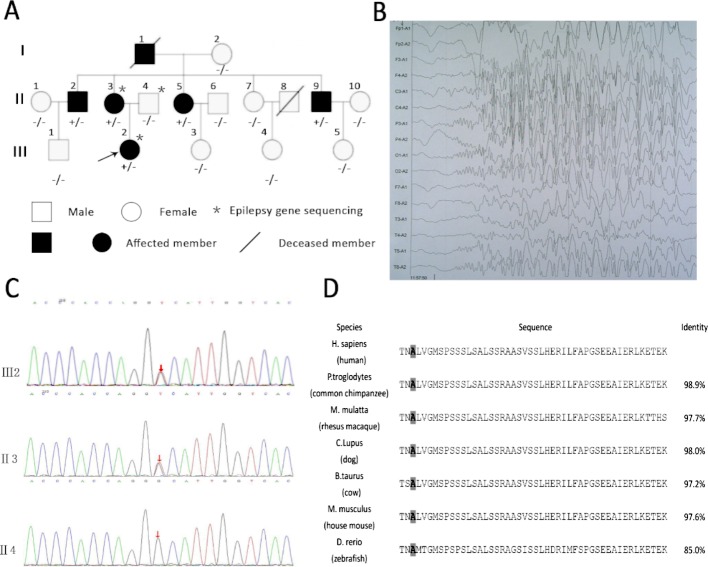
Rare variants in *KIF1A* detected in a family and KIF1A are conserved across species. **(A)** Rare mutation in *KIF1A* detected in a family. **(B)** Electroencephalogram (EEG) of the proband (III2) showing generalized sharp waves. **(C)** The analysis of sequences of affected girl (III2), affected mother (II3), and unaffected father (II4). **(D)** The amino acids coded by variation are conserved across species as showing in the boxed. The last column shows identity with the human protein. The gray boxes highlight the amino acids mentioned in the manuscript.

The family of interest had six affected patients over three generations, which suggests autosomal dominant inheritance (**Figure 1A**). The clinical characteristics of the epilepsy patients in the family are described in **Table 1**. All the affected individuals presented with generalized tonic-clonic seizures without mental retardation, and three of these patients suffered from diabetes mellitus. The seizure onset age of patient III2 was 12 years, and electroencephalogram (EEG) showed generalized sharp waves that were prevalent bilaterally (**Figure 1B**). She started treatment with levetiracetam, which resulted in good control of her seizures. The seizure onset ages of the other patients, namely, II2, II3, II5, and II9, were 18, 16, 21, and 24 years, respectively. None of these individuals had received standard anti-epileptic therapy. Patient II9 presented an abnormal brain CT scan due to ischemic stroke, and the CT scans of the brains of the other patients were normal.

**Table 1:** Clinical characteristics of epilepsy patients in the family.

Pedigree reference	Gender	Age (years)	Age at seizure onset (years)	Seizure frequency	Application of AEDs	Comorbidity
III2	Female	21	12	4–5/year	LEV, 1,000 mg	—
II2	Male	57	18	Once/month	CBZ, 300 mg	Diabetes, hypertension
II3	Female	54	16	1–2/year	VPA, 1,000 mg	—
II5	Female	51	21	6–8/year	VPA, 750 mg LEV, 750 mg	Diabetes, hypertension
II9	Male	35	24	Once/week	OXC, 600 mg	Hypertension, diabetes, stroke

We first performed customized sequencing of epilepsy-related genes of two epilepsy patients (III2 and II3) and one unaffected individual (II4) and identified a possible causal variant (c.1190C > A, p. Ala397Asp) on the neck-coiled coil of *KIF1A* in heterozygosis. The frequency of this mutation in the Genome Aggregation Database (http://gnomad.broadinstitute.org) is 0.000008133. Sanger sequencing of this gene in all members of this family was then performed to assess the segregation of *KIF1A* mutations. This rare mutation was found in all affected family members and was not detected in the unaffected family members (**Figure 1C**). In addition, the mutation was presumed to be damaging by PolyPhen-2 and MutationTaster.

**Figure 2:**
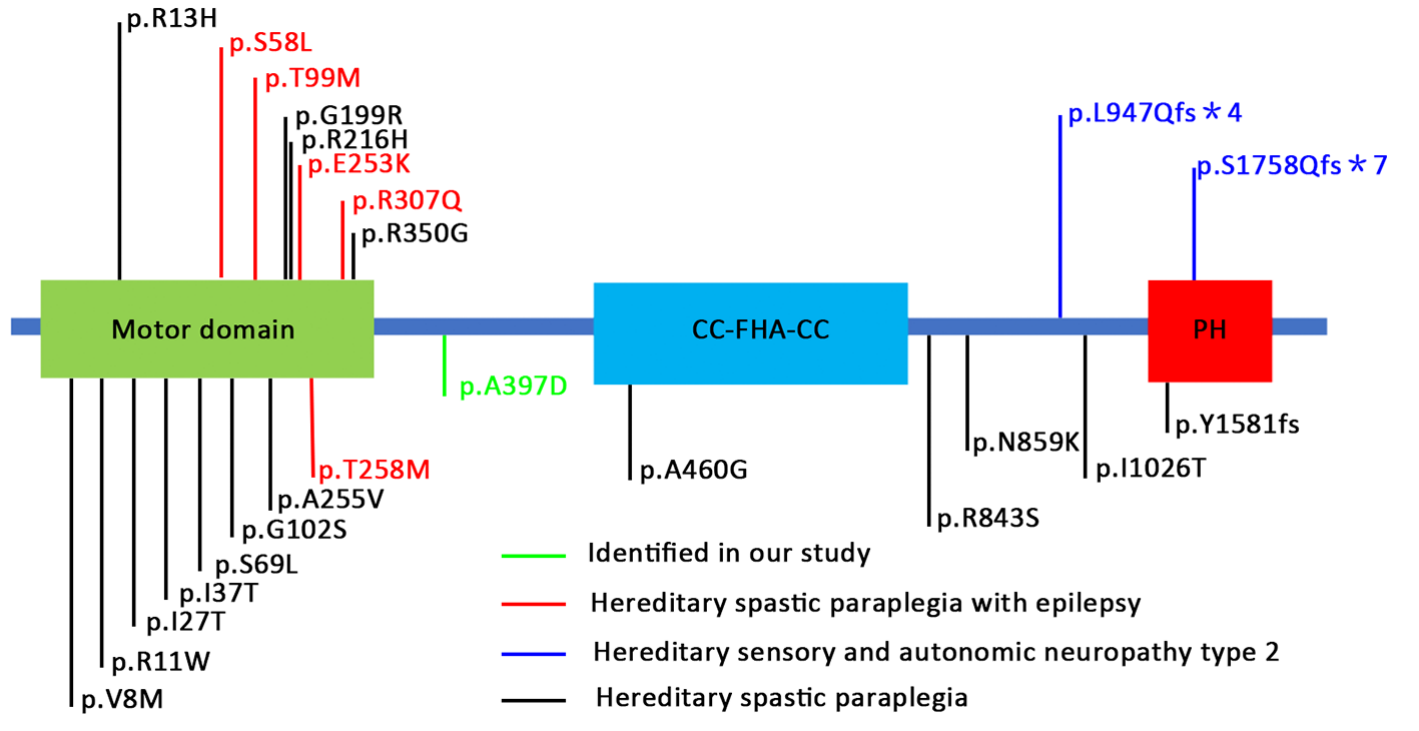
Schematic representation of KIF1A protein and locations of mutations in human KIF1A associated with various neuronal disorders. These mutations are distributed in various locations and domains of the KIF1A subunit protein peptide (Niwa et al., 2016; Iqbal et al., 2017 **; Riviere et al., 2011; Chiba et al., 2019).

The location of the identified mutation in KIF1A is strongly conservation between all species [(**Figure 1D**) www.ncbi.nlm.nih.gov/homologene]. As shown in **Figure 2**, many point mutations in KIF1A have been identified to date, and these mutations have been associated with various neuropathies.

### Effect of Mutant *KIF1A* on the Intrinsic Excitability of Primary Cultured Neurons

Prior to using the primary cultured neurons in subsequent experiments, the expression of the wild-type and mutant KIF1A proteins in the neurons was confirmed by western blot and immunofluorescence. The western blot results suggest that the mutant and wild-type proteins were expressed equally in the primary cultured neurons (**Supplementary Figures S1A, B**). Also, we revealed the wild type protein and mutant protein expressed equally in soma and nerites (**Supplementary Figures S1C, D**).

**Figure 3:**
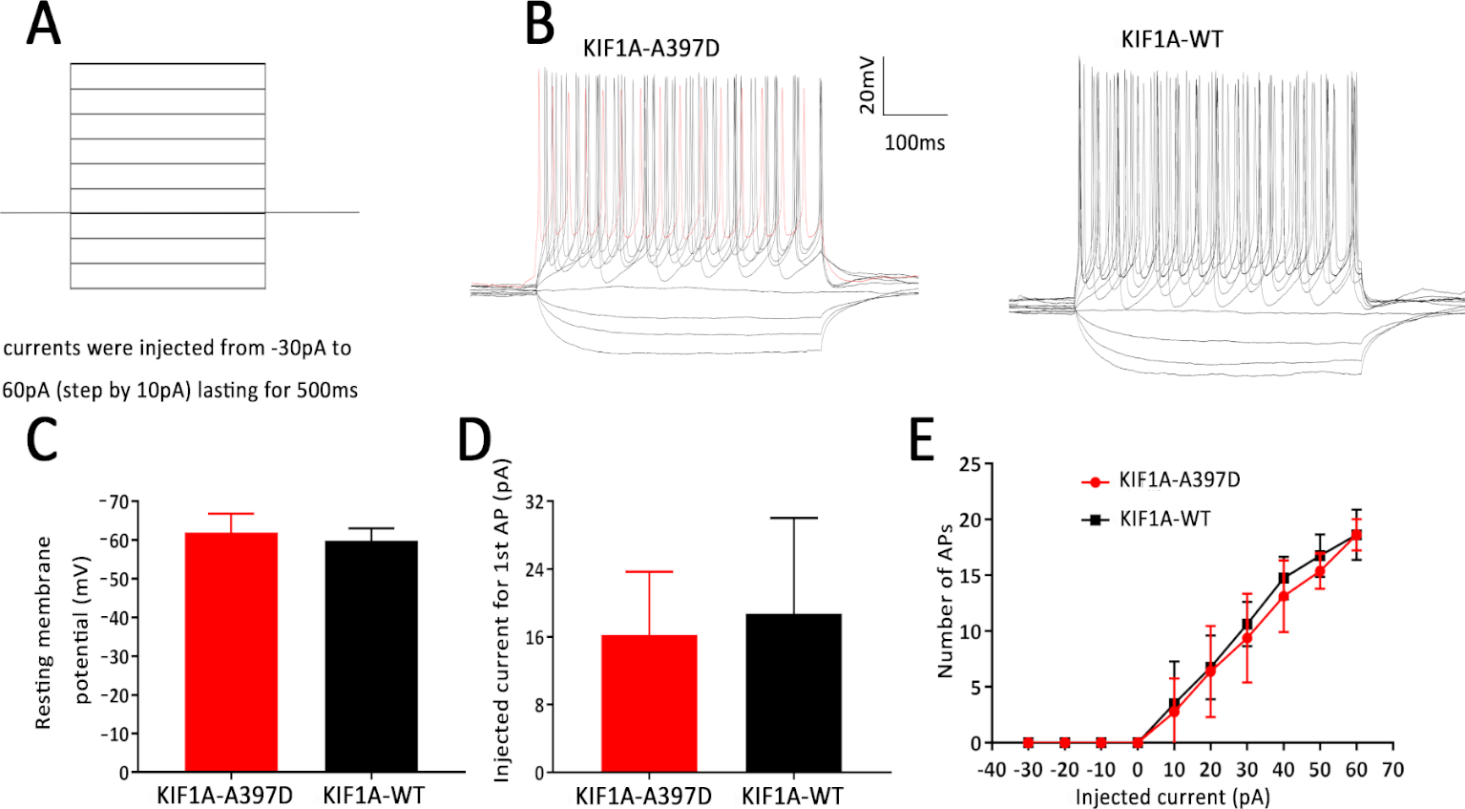
Effect of mutant *KIF1A* on the intrinsic excitability of primary cultured neurons. **(A)** Recording paradigm of passive excitability in the excitatory cultured neurons. **(B)** Examples of the AP responses to superimposed current steps recorded from primary cultured GFP-positive hippocampal neurons transfected with the mutant or wild-type KIF1A plasmid. **(C)** Resting membrane potential of the examined neurons from two groups (n = 8 neurons in each group, Student's *t* test). **(D)** Injected currents used to induce the first spikes (n = 8 neurons in each group, Student's *t* test). **(E)** Number of APs induced by the injected currents in the primary cultured GFP-positive hippocampal neurons transfected with the mutant or wild-type KIF1A plasmid (n = 8 neurons in each group, two-way ANOVA).

In general, increased neuronal firing is due to intrinsic excitability or altered synaptic transmission. Therefore, we tested the intrinsic excitability of the neurons through whole-cell patch-clamp recordings of the primary cultured neurons. The primary cultured neurons were transfected with the indicated plasmid at DIV7, and the whole-cell recording was performed at DIV14. We first checked the resting membrane potential of each neuron and found no difference between the WT and mutant groups (**Figure 3C**). A depolarizing current of 500 ms was then applied in the current clamp mode starting from −30 pA and at increments of 10 pA to induce neuronal firming (**Figure 3A**). We analyzed the injected current intensity between the two groups and found no difference (**Figure 3D**). The action potential recordings revealed that the number of action potentials induced by the injected currents was unaffected (**Figures 3B, E**). In conclusion, mutant *KIF1A* did not affect the intrinsic excitability of neurons.

### Effect of Mutant *KIF1A on* Miniature Excitatory Post-Synaptic Currents and Miniature Inhibitory Post-Synaptic Currents in Primary Cultured Neurons

**Figure 4:**
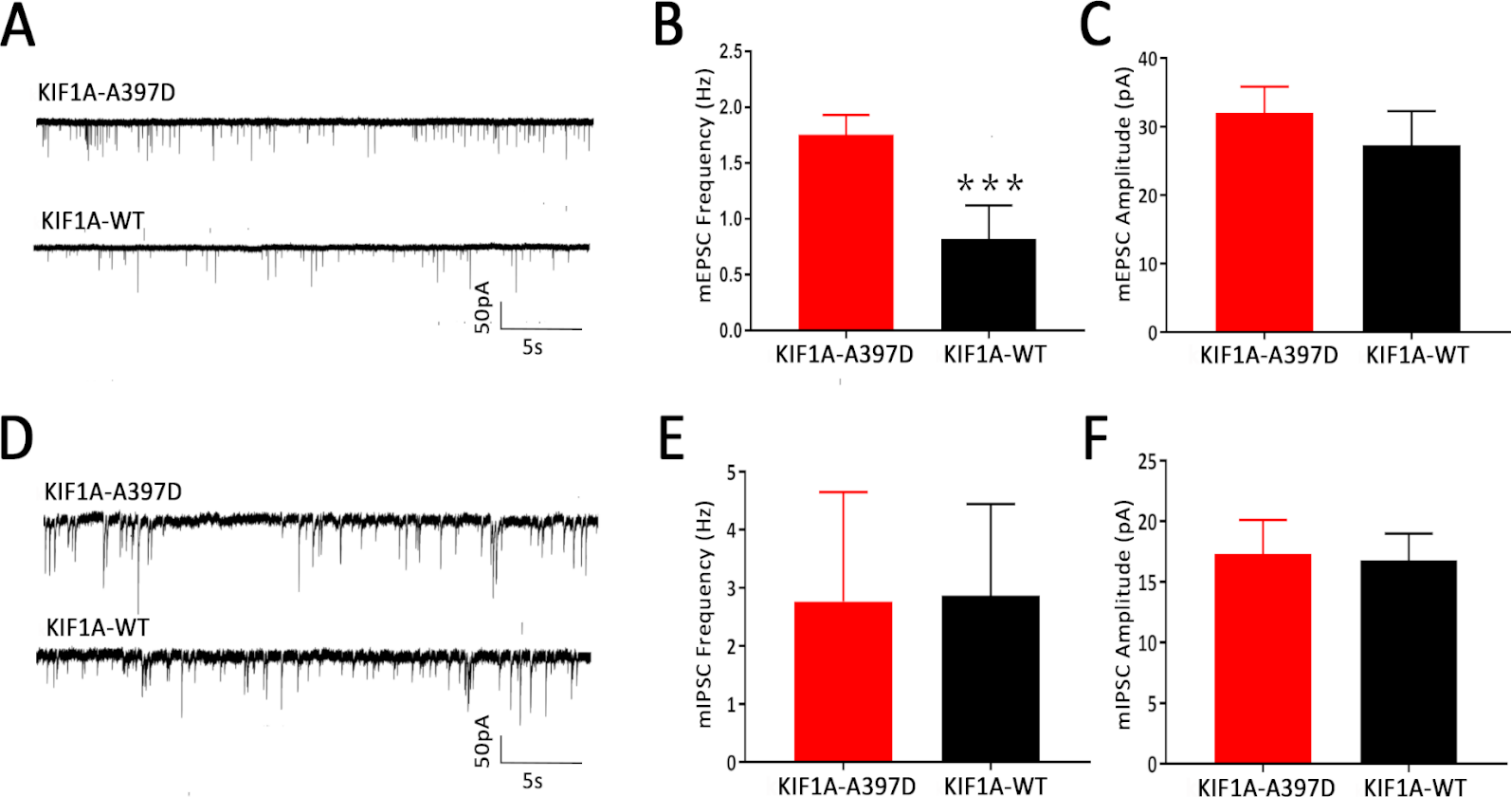
Effect of mutant *KIF1A* on the miniature excitatory post-synaptic currents (mEPSCs) and miniature inhibitory post-synaptic currents (mIPSCs) of primary cultured neurons. **(A)** Representative trace of mEPSCs from cultured hippocampal neurons transfected with the mutant or wild-type KIF1A plasmid at a holding potential of −70 mV. **(B, C)** Bar graph analysis of the mEPSC frequency and amplitude (n = 8 neurons in each group, ***p < 0.001, Student's *t* test). **(D)** Representative trace of mIPSCs from cultured hippocampal neurons transfected with the mutant or wild-type KIF1A plasmids at a holding potential of −70 mV. **(E, F)** Bar graph analysis of the mIPSC frequency and amplitude [n = 10 in the mutant group and 8 in the wild type (WT) group, Student's *t* test].

We did not find any difference in intrinsic excitability between the WT and mutant groups and therefore speculate that mutant *KIF1A* might disrupt synaptic transmission. To investigate this hypothesis, we obtained whole-cell patch-clamp recordings of mEPSCs and mIPSCs in neurons at DIV14 that had been transfected with the plasmid at DIV7. Compared with the expression of WT KIF1A, the expression of mutant KIF1A resulted in a significant increase in the frequency of mEPSCs in neurons, whereas the amplitude of mEPSCs did not show a significantly difference between neurons expressing WT and those expressing mutant KIF1A (**Figures 4A–C**). The mIPSC analysis revealed that the frequency and amplitude did not differ between the two neuron groups (**Figures 4D–F**). Taken together, these results indicate that the expression of mutant KIF1A leads to an enhanced excitatory synaptic transmission.

### Effect of Mutant *KIF1A* on Neuronal Development

**Figure 5:**
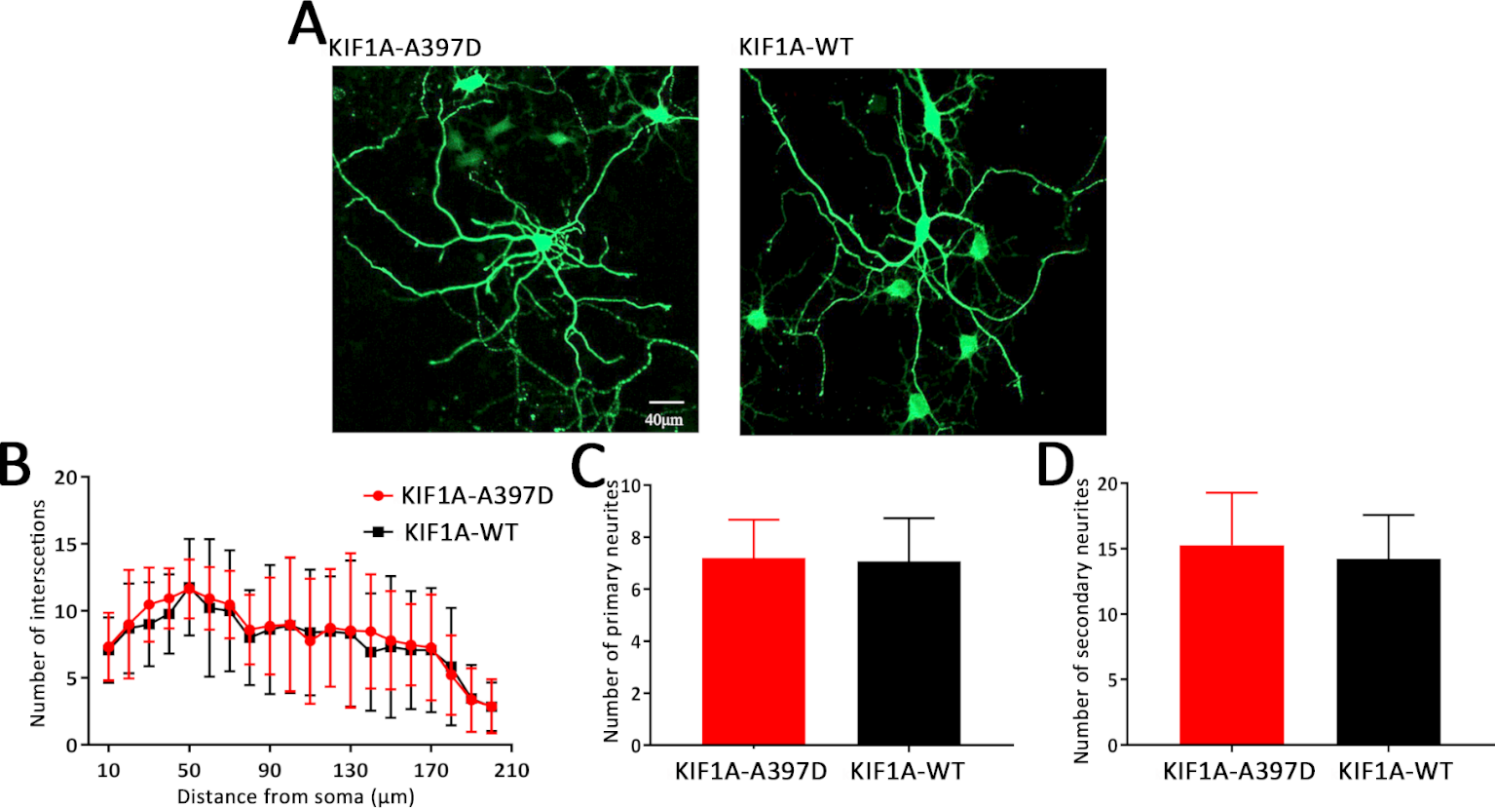
Effect of mutant *KIF1A* on neuronal development. **(A)** Representative image of cultured primary hippocampal neurons obtained through a Sholl analysis. The radius interval between circles was 10 μm per step and ranged from 10 to 210 μm from the center of the neuronal soma. **(B)** Sholl analysis of neurons expressing the mutant (n = 15 neurons) or wild-type protein (n = 13 neurons) (two-way ANOVA). **(C, D)** Number of primary and secondary neurites of neurons expressing the mutant (n = 15 neurons) or wild-type protein (n = 13 neurons) (Student's *t* test).

Previous studies have shown that KIF1A is a neuron-specific expressed protein (Okada et al., 1995) and have demonstrated that knockout of *KIF1A* in mice results in death within 24 h after birth (Yonekawa et al., 1998). We thus hypothesize that *KIF1A* plays an important role in neuronal development and that mutant *KIF1A* might be involved in neuronal development. To test this hypothesis, we transfected primary cultured neurons with a plasmid encoding either WT or mutant *KIF1A* at DIV7 and fixed these cells at DIV10. The resulting neuronal branching was analyzed through Sholl analysis, and surprisingly, no difference in neuronal branching was found between the WT and mutant neurons (**Figures 5A, B**). We subsequently measured the number of primary and secondary neurites and found that mutant KIF1A did not affect the number of neurites (**Figures 5C, D**). In brief, mutant *KIF1A* does not affect neuronal development.

### Effect of Mutant *KIF1A* on Excitatory Synapses

**Figure 6:**
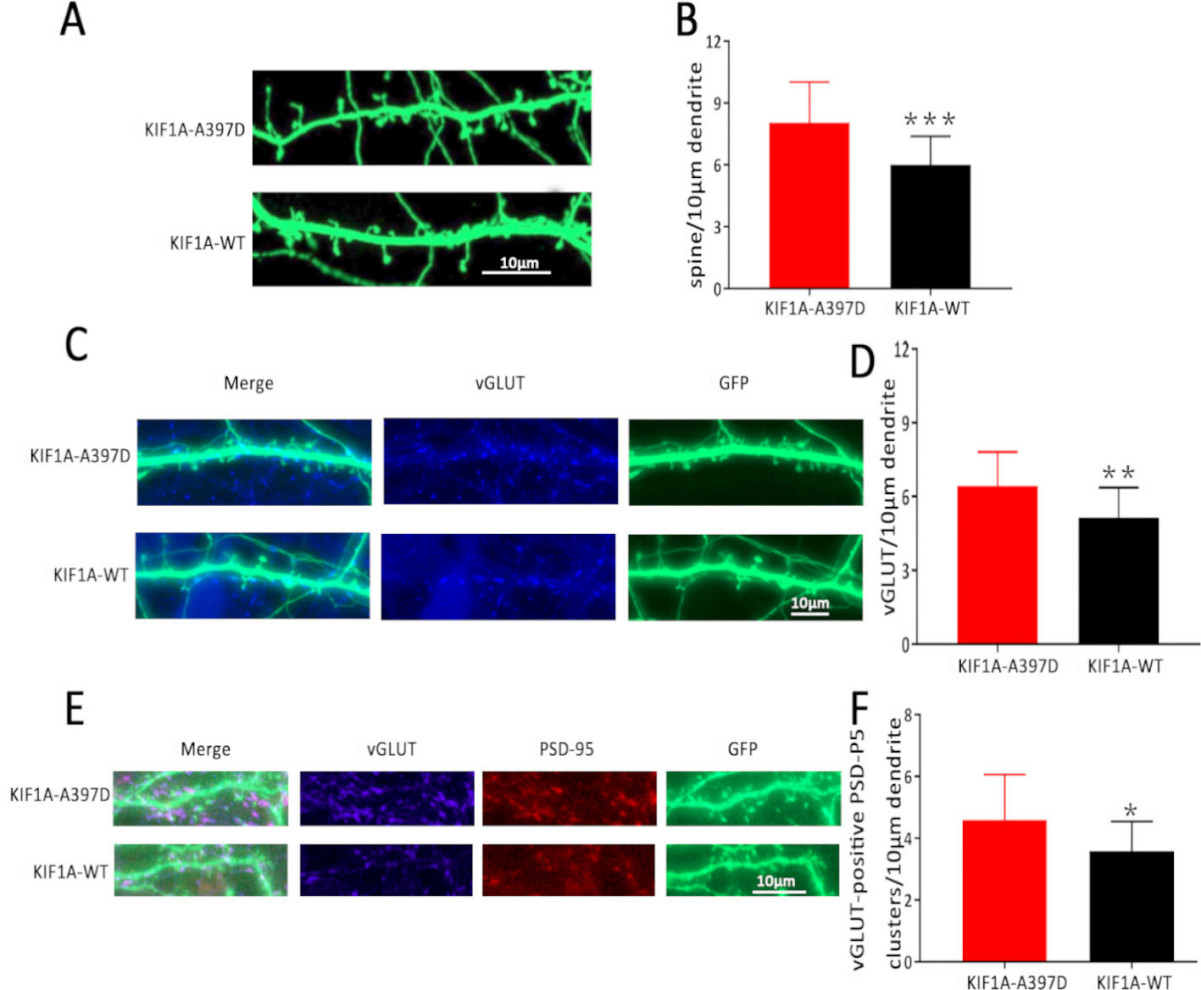
Effect of mutant *KIF1A* on excitatory synapses. **(A)** Representative image of dendritic spines at DIV16 from neurons transfected with the mutant or wild-type KIF1A plasmid. **(B)** Total spines/10 μm of neurons expressing the mutant (n = 26 neurons) or wild-type protein (n = 23 neurons). (***p < 0.001, Student's *t* test). **(C)** Representative image of vGLUT at DIV16 from neurons transfected with the mutant or wild-type KIF1A plasmid. **(D)** Total vGLUT/10 μm of neurons expressing the mutant (n = 24 neurons) or wild-type protein (n = 21 neurons). (**p < 0.01, Student's *t* test). **(E)** Representative image of excitatory synapses (vGLUT-positive PSD-95 clusters) at DIV16 from neurons transfected with the mutant or wild-type KIF1A plasmid. **(F)** Total excitatory synapses (vGLUT-positive PSD-95 clusters)/10 μm of neurons expressing the mutant (n = 19 neurons) or wild-type protein (n = 18 neurons). (*p < 0.05, Student's *t* test).

KIF1A is essential for synaptogenesis in the hippocampus (Kondo et al., 2012). In our study, the expression of mutant *KIF1A* resulted in a significant increase in the frequency of mEPSCs in neurons compared with that observed with WT KIF1A. The increased frequency of mEPSCs might be due to increased presynaptic vesicle release probability, and the increased number of excitatory synapses per neuron is also responsible for the observed increase in the frequency of mEPSCs. Dendritic spines contain the majority of the excitatory synapses of hippocampal pyramidal neurons, and changes in the spine density lead to alterations in the mEPSC frequency and are associated with aberrations in synaptic plasticity. To determine whether the mutant *KIF1A* affects the dendritic spines, we transfected neurons with a plasmid construct encoding the WT or mutant KIF1A at DIV7 and fixed these neurons at DIV16. Consistent with our hypothesis, the neurons transfected with mutant KIF1A exhibited a significantly higher density of dendritic spines and vGLUT compared with the neurons transfected with WT KIF1A (**Figures 6A–D**). To further verify the excitatory synapse formation, vGLUT-positive and PSD-95 clusters were examined using double immunofluorescence staining (**Figures 6E, F**), our results suggest that mutant KIF1A is responsible for the observed increase in the excitatory synaptic density.

### Effect of Mutant *kif1aa* on the Behavior of and Local Field Potentials in Zebrafish

We subsequently investigated the functional consequence of mutant kif1a *in vivo* by establishing a zebrafish model. Zebrafish have two *kif1a* homologue genes termed *kif1aa* and *kif1ab* that encode two protein isoforms, Kif1aa and Kif1ab, which show 85 and 80% identity to human KIF1A, respectively. The mutant alanine in the affected patients corresponds to A433 in Kif1aa and A410 in Kif1ab. Both of these genes have the same function in neurons, and *kif1aa*, which is also located on chromosome 2, and Kif1aa share higher (85%) similarity with human KIF1A. Therefore, we selected zebrafish *kif1aa* for our zebrafish experiment.

**Figure 7:**
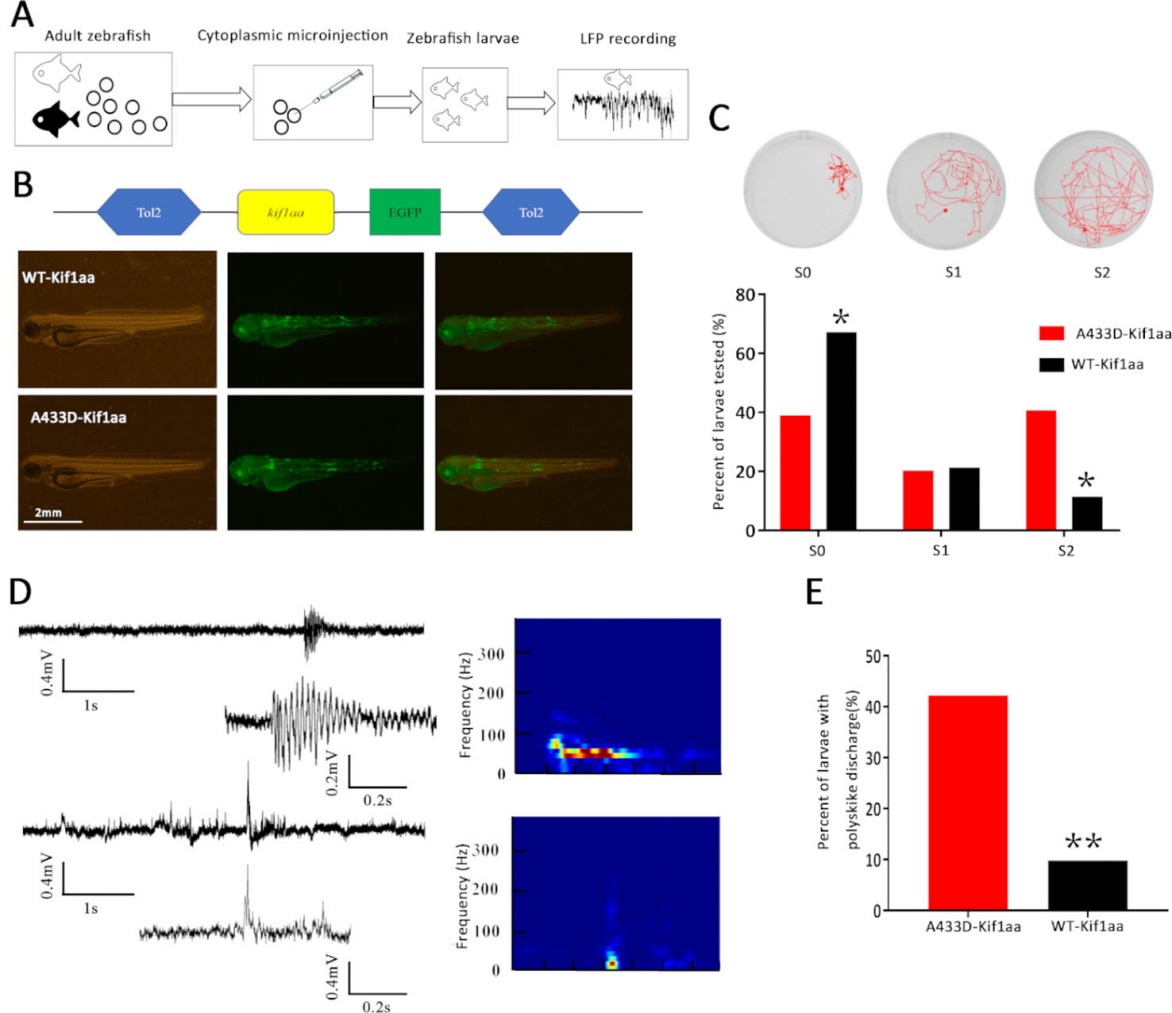
Overexpression of mutant Kif1aa causes abnormal behavior and epileptiform discharges in transgenic zebrafish larvae. **(A)** Graphic representation of the experimental protocol used for the zebrafish studies. **(B)** Representative images of zebrafish larvae microinjected with A433D-kif1aa (n = 64) or wild type (WT)-kif1aa (n = 61) selected for local field recordings. The distribution of the labeled protein is shown by green fluorescence. **(C)** Seizure behaviors of zebrafish larvae. The sample locomotion tracking plots are shown on the top (S0, baseline activity; S1, small increase in swim activity; and S2, large increase in movement. The bar plot shows the percentage of tested larvae recorded during locomotion tracking (*p < 0.05, χ^2^ test). **(D)** Representative trace of spontaneous epileptiform activity recorded from zebrafish larvae. The top trace represents a typical epileptiform pattern observed in gap-free recordings. The bottom trace shows a high-resolution magnification of the selected epileptiform events, and the frequency spectrum corresponding to the selected local field potentials (LFPs) is shown on the right. Color scale: blue (low magnitude) to red (high magnitude). **(E)** Percentage of larvae with abnormal epileptiform activity after overexpression of A433D (n = 64) or WT Kif1aa (n = 61). (**p < 0.01, χ^2^ test).

To establish the zebrafish model, we cloned mutant and WT *kif1aa* into the tol2 expression vector (WT *kif1aa*-Tol2 or A433D *kif1aa*-Tol2) and evaluated the expression of Kif1aa in zebrafish larvae using GFP. Mutant (A433D *kif1aa*-Tol2) or WT *kif1aa* (WT *kif1aa*-Tol2) was introduced into embryos at the one-cell stage by cytoplasmic microinjection (**Figure 7A**). Three days later, neither WT nor mutant zebrafish exhibited gross dysmorphologies (**Figure 7B**). Normal-looking zebrafish larvae displaying a touch response at 5 d.p.f. were selected for behavior monitoring. These zebrafish larvae were placed in a well of a 24-well falcon plate, and the motors of the freely swimming fish were observed using a stereomicroscope. The results showed that 67.2% of the WT larvae displayed normal behavior that could be characterized as S0 (baseline activity), and only 11.5% of the WT larvae exhibited abnormal behaviors that could be characterized S2 (large increase in movement). The analysis of the mutant larvae revealed that 40.6% displayed excessive motor activity that could be characterized as S2, and the percentage of larvae showing baseline activity (S0) was 39.0%, which was significantly lower than that found for the WT larvae (**Figure 7C**). To determine whether the missense mutation in Kif1aa resulted in excessive brain electrophysical activity, we obtained local field potential recordings from the zebrafish tectum at 5 d.p.f. and observed spontaneous epileptiform activity (polyspike discharges) in 42.2% of mutant larvae and 9.8% of WT larvae (this difference was significant) (**Figures 7D, E**).

## Discussion

Although it has been confirmed that most patients with epilepsy have genetic epilepsy, the transmission of the epilepsy phenotype is clearly autosomal dominant or autosomal recessive. However, the genetic etiology of generalized epilepsy in the majority of patients is unknown. In recent decades, extensive use of high-throughput sequencing in recent years has confirmed that a large amount of gene alterations might be responsible for epileptogenesis (Helbig et al., 2016; McTague et al., 2016). In our study, we identified a rare mutation of *KIF1A* in a family with six affected patients over three generations using a panel of epilepsy-related genes and Sanger sequencing. We first investigated the possible mechanism in primary cultured neurons and found that mutant KIF1A increased the density of excitatory synapse, which indicates that the mutation might be a gain-of-function mutation. A functional test using zebrafish showed that the mutation resulted in epileptic activity.

KIF1A, a kinesin-3 member, is a unique monomeric neuron-specific motor protein. is mainly involved in the anterograde transport of synaptic vesicle proteins in axons, such as synaptotagmin, synaptophysin, and Rab3A (Okada et al., 1995). Several studies have shown that KIF1A is also responsible for neuron migration and synaptic plasticity (McVicker et al., 2016; Stucchi et al., 2018). KIF1A has four sections: a motor domain, a neck coiled-coil region, a CC-FHA-CC domain, and a globular tail/pleckstrin homology domain (Huo et al., 2012; Lee et al., 2015). The motor domain mainly includes a nucleotide catalytic binding site and a microtubule-binding site. The neck coiled-coil region, which is attached to the motor domain, is very flexible, undergoes different conformational changes at different nucleotide-binding states, and produces tension through docking and separation from the motor domain. The CC-FHA-CC domain is located in the middle of the isoform. The end of the stalk region contains the globular tail/pleckstrin homology domain, which recognizes vesicles and membranous organelles (Hirokawa et al., 2009) Thus far, limited studies have investigated the role of KIF1A in the origin of epilepsy. Previous studies have reported that several patients with mutations in *KIF1A* present recurrent seizures; however, these studies investigated only the correlation of neuropathy and brain malformation with mutations in *KIF1A* (Esmaeeli Nieh et al., 2015; Lee et al., 2015; Hotchkiss et al., 2016; Megahed et al., 2016; Cheon et al., 2017; Demily et al., 2018). All of these reported mutations accompanied by recurrent seizures were located in the motor domain of KIF1A and resulted in decreased motor activity. In our study, we found a missense mutation located in the neck coiled-coil region, and the maintenance of epileptic susceptibility observed with this mutant KIF1A was mainly an outcome of enhanced excitatory synaptic plasticity rather than individual properties. We examined the mEPSCs and mIPSCs of primary cultured neurons and found that only the frequency of mEPSCs was altered in the mutant group, which indicates effects on synaptogenesis.

To confirm this finding, we investigated synaptogenesis in neurons. Dendritic spines are the synaptic component in the majority of excitatory neurons, and the observed increase in spine density and vGLUT is consistent with the increased frequency of mEPSCs. To some extent, spine alterations signify the neuronal network dynamics (Cooke and Woolley, 2009). Previous studies have demonstrated that aberrant alterations in the dendritic spine density are commonly observed in brain samples from epilepsy patients and epilepsy animal models (Jiang et al., 1998; Wong and Guo, 2013). Dendritic spine abnormalities might increase the hyperexcitable circuits or intrinsic properties of neurons that might cause seizures, and seizures also result in damage to dendrites and dendritic spines, which might contribute to progressive recurrent seizures, mental disorders, memory disturbances, and other neurological deficits in epilepsy patients (Jimenez-Mateos et al., 2015; Awad et al., 2016; Carter et al., 2017)

Previous study suggest that KIF1A mutations that cause hereditary spastic paraplegia are loss-of-function mutations that decrease motility (Esmaeeli Nieh et al., 2015). However, not all mutations are loss-of-function. Recently, Chiba *et al*. revealed gain-of-function mutations in KIF1A, V8M, A255V, and R350G that cause overactivation of KIF1A motor activity *in vitro*. KIF1A (V8M), KIF1A (A255V), and KIF1A (R350G) increased the landing rate (20-fold or 10-fold higher than the WT), and the velocity of KIF1A (V8M) and KIF1A (R350G) was ∼2- and 3-fold faster, respectively, than WT KIF1A (Chiba et al., 2019). Other mutations (E412K, G598R, and E612K) showed with similar phenomena (Niwa et al., 2016). However, the molecular mechanism of these changes remains unclear, and the authors speculate that these mutations could conceivably alter the motor enzymatic rate or lead to varying degrees of autoinhibition release. Interestingly, the abovementioned gain-of-function mutation results in a reduced synaptic size in *Caenorhabditis elegans*. In clinical presentation, the phenotype of gain-of-function mutant individuals was milder than in loss-of-function mutant individuals (Klebe et al., 2012; Chiba et al., 2019). The gain-of-function mutations located in the motor domain increased the landing rate or velocity. In our study, the KIF1A (A397D) mutation, which is located in the neck coiled-coil (mapped between the motor domain and CC-FHA-CC domain), was also revealed as a gain-of-function mutation (increased excitatory synapse). Therefore, we speculate that the KIF1A (A397D) mutant may have caused increased excitatory synaptic density and seizure activity by increasing the landing rate and/or velocity, but these increases are not sufficient for triggering other neuropathies [e.g., spastic paraplegia (SPG) and intellectual disability].

To validate that mutant KIF1A is a gain-of-function mutation *in vivo*, we constructed overexpression transgenic zebrafish using the tol2 vector. Zebrafish have two *kif1a* homologous genes, *kif1aa* and *kif1ab*. The *kif1aa* gene is located on chromosome 2, and its encoded protein, Kif1aa, shares 85% identity with human KIF1A. Therefore, we selected the corresponding residue A433D in zebrafish Kif1aa for the zebrafish experiments. Interestingly, our functional experiment demonstrated that the overexpression of Kif1aa in the vertebrate *in vivo* system results in abnormal seizure-like behavior and epileptiform-like discharges.

In conclusion, our data provide evidence demonstrating that the kinesin superfamily member *KIF1A* is involved in epileptogenesis. The identification of other epilepsy patients with mutations in this gene should further confirm the role of *KIF1A* and might provide further insights into the full clinical spectrum. The functional-level results indicated that the mutation in the neck linker of KIF1A resulted in increased dendritic spines, which might be a main cause of aberrant neuronal circuits in the brain.

## Data Availability Statement

The datasets generated for this study can be found in NCBI GenBank accession MN897723.

## Ethics Statement

The studies involving human participants were reviewed and approved by Ethics Committee of Chongqing Medical University. The patients/participants provided their written informed consent to participate in this study. The animal study was reviewed and approved by Ethics Committee of Chongqing Medical University. Written informed consent was obtained from the owners for the participation of their animals in this study. Written informed consent was obtained from the individual(s) for the publication of any potentially identifiable images or data included in this article

## Author Contributions

YG, YM, XW, and XT conceived the project and designed the experiments. YG, YC, MY, XX, ZL, JM, HC, YH, YM, and XT performed the experiments. YG, XW, and XT wrote the manuscript. All authors revised and approved the final version of the manuscript.

## Funding

This work was supported by grants from the National Natural Science Foundation of China (81671301, 81901332, and 81701279), Chongqing Nature Science Foundation Project (csts2019jcyj-msxmX0184) and Cultivating Fund of the First Affiliated Hospital of Chongqing Medical University (PYJJ2019-201 and PYJJ2018-11).

## Conflict of Interest

The authors declare that the research was conducted in the absence of any commercial or financial relationships that could be construed as a potential conflict of interest.
