# Supplementary material for: A Rare KIF1A Missense Mutation Enhances Synaptic Function and Increases Seizure Activity
Source: Front Genet. 2020 Feb 27;11:61. doi: 10.3389/fgene.2020.00061 (PMC7056823; doi:10.3389/fgene.2020.00061)
Supplement: Supplementary file 1 [file Table_1.docx]

Supplementary table 1: List of genes analyzed in customized epilepsy-related gene

| *ABAT* | *BCKDK* | *CTSA* | *FASTKD2* | *HNF1B* | *MDGA2* | *OPHN1* | *RAB3GAP1* |
| --- | --- | --- | --- | --- | --- | --- | --- |
| *ABCC2* | *BCS1L* | *CTSD* | *FCGR2B* | *HNRNPH1* | *ME2* | *RAF1* | *PAFAH1B1* |
| *ABCC8* | *BOLA3* | *CTSF* | *FGD1* | *HNRNPU* | *MECP2* | *PAH* | *RANBP2* |
| *ACADSB* | *BRAF* | *CUL4B* | *FGF8* | *HP* | *MED12* | *PAK3* | *RARS2* |
| *ACOX1* | *BSN* | *CYB5R3* | *FGFR3* | *HPD* | *MEF2C* | *PANK* | *RBFOX1* |
| *ACTB* | *BTD* | *CYP2R1* | *FH* | *HRAS* | *MEF2C* | *PAX6* | *RBFOX2* |
| *ACY1* | *TWNK* | *CYP2U1* | *FKRP* | *HSD17B10* | *MGAT2* | *PC* | *RBFOX3* |
| *COQ8A* | *C12orf65* | *D2HGDH* | *FKTN* | *HSD17B4* | *MLC* | *PCDH19* | *RELN* |
| *ADK* | *C4A* | *DAO* | *FLNA* | *HTR2A* | *MMACHC* | *PCNT* | *RFT1* |
| *ADSL* | *CACNA1A* | *DAOA* | *FOLR1* | *HTT* | *MOCS1* | *PDHA1* | *RNASEH2A* |
| *AFG3L2* | *CACNA1H* | *DBH* | *FOXG1* | *HYAL1* | *MOCS2* | *PDHA* | *RNASEH2B* |
| *AGA* | *CACNB4* | *DBT* | *FOXP2* | *IDH1* | *MOCS3* | *PDSS1* | *RNASEH2C* |
| *AHI1* | *CACNG2* | *DCX* | *FOXRED1* | *IDS* | *MOGS* | *PDSS2* | *ROGD1* |
| *AKT1* | *CASK* | *DDC* | *FUCA1* | *IDUA* | *MPC1* | *PEX1* | *RPGRIP1L* |
| *ALDH4A1* | *CASR* | *DDOST* | *GABBR2* | *IER3IP1* | *MPDU1* | *PEX10* | *RPIA* |
| *ALDH5A1* | *CC2D2A* | *DEPDC5* | *GABRA1* | *IFNG* | *MPI* | *PEX12* | *RTN4R* |
| *ALDH7A1* | *CDH13* | *DGKD* | *GABRA6* | *IL6* | *MR1* | *PEX13* | *RYR1* |
| *ALG1* | *CDH9* | *DHCR7* | *GABRB2* | *INPP5E* | *MTHFR* | *PEX14* | *RYR3* |
| *ALG11* | *CDKL5* | *DHFR* | *GABRB3* | *INS* | *MTOR* | *PEX16* | *SAMHD1* |
| *ALG12* | *CEP152* | *DIAPH3* | *GABRD* | *IQSEC2* | *MTR* | *PEX19* | *SCARB2* |
| *ALG13* | *CEP290* | *DISC1* | *GABRG2* | *KCNA1* | *MTRR* | *PEX2* | *SCN1A* |
| *ALG2* | *CHD2* | *DLD* | *GALC* | *KCNB1* | *NAGLU* | *PEX26* | *SCN1B* |
| *ALG3* | *CHI3L1* | *DMPK* | *GALNS* | *KCNH5* | *NDE1* | *PEX3* | *SCN2A* |
| *ALG6* | *CHRNA2* | *DNAJC5* | *GAMT* | *KVNJ1* | *NDN* | *PEX5* | *SCN4A* |
| *ALG8* | *CHRNA3* | *DNAJC6* | *GATM* | *LCNJ10* | *NDUFA1* | *PEX6* | *SCN8A* |
| *ALG9* | *CHRNA4* | *DNASE1* | *GBA* | *KCNJ11* | *NDUFA11* | *PEX7* | *SCN9A* |
| *AMACR* | *CHRNA5* | *DNM1* | *GCDH* | *KCNMA1* | *NDUFA2* | *PGK1* | *SCO2* |
| *AMER1* | *CHRNA7* | *DOCK6* | *GCK* | *KCNQ1* | *NDUFAF1* | *PGM1* | *SDHA* |
| *AMT* | *CHRNB2* | *DOCK7* | *GCSH* | *KCNQ2* | *NDUFAF2* | *PHF6* | *SEROINI1* |
| *APOL2* | *CLCN2* | *DOLK* | *GFAP* | *KCNQ3* | *NDUFAF3* | *PHGDH* | *SETBP1* |
| *APOL4* | *CLCN4* | *DPAGT1* | *GLB1* | *KCNT1* | *NDUFAF4* | *PIGA* | *SGCE* |
| *APP* | *CLCNKA* | *DPM1* | *GLDC* | *KCTD7* | *NDUFAF5* | *PIGL* | *SGSH* |
| *APTX* | *CLCNKB* | *DPM3* | *GLRA1* | *KDM5C* | *NDUFB3* | *PIGV* | *SHANK3* |
| *ARG1* | *CLN3* | *DPYD* | *GLRB* | *KIF11* | *NDUFS1* | *PLA2G6* | *SHH* |
| *ARHGAP31* | *CLN5* | *DRD2* | *GLUD1* | *KIF1A* | *NDUFS2* | *PLCB1* | *SHOC2* |
| *ARHGEF9* | *CLN6* | *DRD3* | *GLUL* | *KMT2D* | *NDUFS3* | *PLP1* | *SIX3* |
| *ARL13B* | *CLN8* | *DTNBP1* | *GNE* | *KRAS* | *NDUFS4* | *PMM2* | *SLC13A5* |
| *ARSA* | *CNTN5* | *EBP* | *GNPTAB* | *KRIT1* | *NDUFS6* | *PNKD* | *SLC16A2* |
| *ARSB* | *CNTNAP2* | *ECM1* | *GNPTG* | *L2HGDH* | *NDUFS7* | *PNKP* | *SLC17A5* |
| *ARSE* | *COA5* | *EEF1A2* | *GNS* | *LAMA2* | *NDUFS8* | *PNPO* | *SLC19A3* |
| *ARX* | *COG1* | *EFHC1* | *GOSR2* | *LARGE1* | *NDUFV1* | *POLG* | *SLC1A3* |
| *ASAH1* | *COG4* | *EHMT1* | *GPC3* | *LBR* | *NDUFV2* | *POMGNT1* | *SLC20A2* |
| *ASPA* | *COG5* | *EIF2B1* | *GPHN* | *LGI1* | *NEDD4L* | *POMT1* | *SLC25A15* |
| *ATIC* | *COG6* | *EIF2B2* | *ADGRG1* | *KGR4* | *NEU1* | *POMT2* | *SLC25A19* |
| *ATN1* | *COG7* | *EIF2B3* | *ADGRV1* | *LIAS* | *NF1* | *PPOX* | *SLC25A22* |
| *ATP13A4* | *COG8* | *EIF2B4* | *GRIA3* | *LIG4* | *NGLY1* | *PPT1* | *SLC26A4* |
| *ATP1A2* | *COL18A1* | *EIF2B5* | *GRIN1* | *LMX1B* | *NHLRC1* | *PQBP1* | *SLC2A1* |
| *ATP1A3* | *COL4A1* | *ELP4* | *GRIN2A* | *LRPPRC* | *NHS* | *PRICKLE1* | *SLC35A1* |
| *ATP2A2* | *COMT* | *EMX2* | *GRIN2B* | *MAGI1* | *NID2* | *PRICKLE2* | *SLC35A2* |
| *ATP5A1* | *COQ2* | *EPB41L1* | *GSS* | *MAGI2* | *NOTCH3* | *PROC* | *SLC35C1* |
| *ATP6AP2* | *COQ9* | *EPHB2* | *GUSB* | *MAGT1* | *NPC1* | *PRODH* | *SLC46A1* |
| *ATP7A* | *COX14* | *EPM2A* | *GYS1* | *MAN1B1* | *NPC2* | *PRRT2* | *SLC6A8* |
| *ATPAF2* | *COX15* | *ERBB4* | *HAX1* | *MANBA* | *NPHP1* | *PSAP* | *SLC9A6* |
| *ATRX* | *COX6B1* | *ERLIN2* | *HDAC4* | *MAP2K1* | *NR3C1* | *PSAT1* | *SLC9A9* |
| *ATXN10* | *CPA6* | *ETFA* | *HEXA* | *MAP2K2* | *NRAS* | *PTCH1* | *SMC1A* |
| *B4GALT1* | *CPS1* | *ETFB* | *HEXB* | *MAPK10* | *NRXN1* | *PTPN11* | *SMPD1* |
| *BANK1* | *CTP1A* | *ETFDH* | *HFE* | *MBD5* | *NTNG1* | *PTPN22* | *SMS* |
| *BCKDHA* | *CPT2* | *EVC* | *HGSNAT* | *MCCC2* | *NUBPL* | *PUS1* | *SNIP1* |
| *BCKDHB* | *CSTB* | *FADD* | *HLA-DQA1* | *MCOLN1* | *OFD1* | *QDPR* | *SNRPN* |
| *SOS1* | *CTNNA3* | *FASN* | *HLA-DQB1* | *MCPH1* | *OPA1* | *RAB39B* | *SOBP* |
| *SPAST* | *SYNGAP1* | *TRPM6* | *ZDHHC15* | *SUCLA2* | *TMEM67* | *VPS13A* | *TUBGCP6* |
| *SPTAN1* | *SYNJ1* | *TSC1* | *ZEB2* | *SUMF1* | *TMEM70* | *VPS13B* | *TUSC13* |
| *SPTLC2* | *SYP* | *TSC2* | *ZFYVE26* | *SUOX* | *TNK2* | *VRK1* | *TYROBP* |
| *SRD5A3* | *SZT2* | *TSEN2* | *ZNF41* | *STXBP1* | *TMEM216* | *UBE3A* | *ST3GAL5* |
| *SRPX2* | *TACO1* | *TSEN34* | *SURF1* | *TPP1* | *VRK2* | *TBX1* | *STRADA* |
| *ST3GAL2* | *TBC1D24* | *TSEN54* | *SYN1* | *TREM2* | *WDR45* | *TCF4* | *STS* |
| *TBP* | *TUBA1A* | *SYN2* | *TREX1* | *XK* | *TMEM165* |  |  |
